# In silico tissue generation and power analysis for spatial omics

**DOI:** 10.1038/s41592-023-01766-6

**Published:** 2023-03-02

**Authors:** Ethan A. G. Baker, Denis Schapiro, Bianca Dumitrascu, Sanja Vickovic, Aviv Regev

**Affiliations:** 1 Klarman Cell Observatory, Broad Institute of MIT and Harvard, Cambridge, MA USA; 2 Department of Biology, Massachusetts Institute of Technology, Cambridge, MA USA; 3 Laboratory of Systems Pharmacology, Harvard Medical School, Boston, MA USA; 4 Institute for Computational Biomedicine, Faculty of Medicine, Heidelberg University Hospital and Heidelberg University, Heidelberg, Germany; 5 Institute of Pathology, Faculty of Medicine, Heidelberg University Hospital and Heidelberg University, Heidelberg, Germany; 6 School of Mathematics, Institute of Advanced Study, Princeton, NJ USA; 7 Department of Computer Science and Technology, University of Cambridge, Cambridge, UK; 8 New York Genome Center, New York, NY USA; 9 Department of Biomedical Engineering and Herbert Irving Institute for Cancer Dynamics, Columbia University, New York, NY USA; 10 Science for Life Laboratory, Department of Immunology, Genetics and Pathology, Beijer Laboratory for Gene and Neuro Research, Uppsala University, Uppsala, Sweden; 11 Present Address: Genentech, South San Francisco, CA USA

**Keywords:** Statistical methods, Software, Transcriptomics, Transcriptomics

## Abstract

As spatially resolved multiplex profiling of RNA and proteins becomes more prominent, it is increasingly important to understand the statistical power available to test specific hypotheses when designing and interpreting such experiments. Ideally, it would be possible to create an oracle that predicts sampling requirements for generalized spatial experiments. However, the unknown number of relevant spatial features and the complexity of spatial data analysis make this challenging. Here, we enumerate multiple parameters of interest that should be considered in the design of a properly powered spatial omics study. We introduce a method for tunable in silico tissue (IST) generation and use it with spatial profiling data sets to construct an exploratory computational framework for spatial power analysis. Finally, we demonstrate that our framework can be applied across diverse spatial data modalities and tissues of interest. While we demonstrate ISTs in the context of spatial power analysis, these simulated tissues have other potential use cases, including spatial method benchmarking and optimization.

## Abstract (web summary)

This paper presents a statistical framework for power analysis of spatial omics studies, facilitated by an in silico tissue-generation method.

## Main

Tissues are composed of organized communities of diverse cell types, each with distinct morphologies, molecular profiles and cellular neighborhoods. In homeostasis, cells interact to establish and maintain proper tissue function, whereas diseases can disrupt spatial organization in specific ways^1^. Analyzing such patterns is a cornerstone of histopathology, providing a critical means for diagnosis in disease, and a key tool for understanding tissue function. Molecular measurements in situ, especially of RNA and protein markers, enhance the available patterns and aid in mechanistic interpretation.

In recent years, emerging methods, including novel spatial transcriptomics and antibody-based spatial proteomics, have dramatically increased the number of molecules that can be measured in one tissue section (Supplementary Table 1). This has vastly increased the number of possible markers, and in some cases, has allowed the discovery of new biomarkers post hoc^1–3^ in both basic and translational settings^1,3–6^. Current spatial transcriptomics and antibody-based proteomics technologies vary greatly in terms of read-out strategies (next-generation sequencing or imaging), the number of targets that can be probed within a sample (dozens to thousands of pre-selected markers to genome-scale), and resolution of each spatial measurement profiled (single molecule, subcellular, cellular, or supra-cellular), as well as throughput achieved through different workflows. Progress in these novel in situ approaches has enabled the scientific community to profile tissues in great detail^7–21^.

Spatial profiling studies can tackle different key questions, including the association of a specific condition or disease state with particular cell types, cell–cell adjacencies, or higher-order structures in the tissue. To address such questions, scientists need to design experiments, including choosing the number of unique tissue sections (‘samples’) and the number and size of fields of view (FOVs) required to detect spatial patterns at a given confidence level. Each of these choices depends on specific assumptions, such as the organization of the tissue, the type of measurements, variation within and between tissue samples (and classes), and the statistical methods used.

**Fig. 1:**
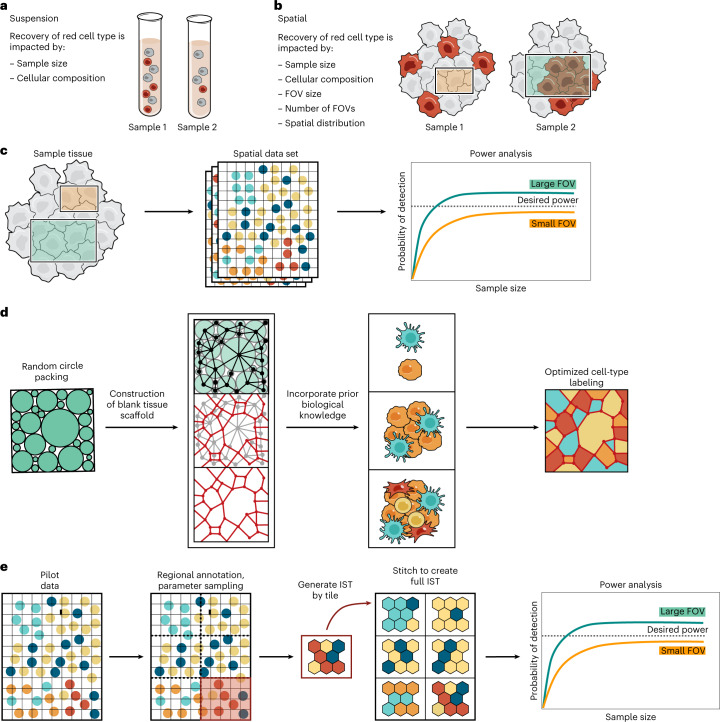
Power analysis framework for spatial omics data. **a**,**b**, Features impacting power to detect cell types in single-cell (**a**) and spatial genomics (**b**) experiments. **c**, Use of spatial data sets for retrospective power analysis. Different sizes of FOVs (squares, left) from an existing spatial data set are sampled, and their data (middle) is used to conduct a statistical analysis. The results (right) are used to calculate the probability of detection of a desired feature (*y* axis, right) when using smaller (orange) or larger (green) FOVs. Dashed line, desired threshold. **d**, Generation of ISTs. From left: our method generates a blank tissue scaffold using a random-circle-packing algorithm (two left panels), and prior biological knowledge is used to optimize cell-type assignments on the tissue scaffold (second from right), followed by visualization with Voronoi diagrams (right). **e**, IST generation of complex or large tissues by regional annotations from pilot data. Pilot data (left) are used to assign regional annotations (second left), and spatial parameters are estimated for each region separately. The region-specific parameters are used to generate IST tiles, which are stitched together to create a full IST (second from right), followed by analysis, for example to compare the sampling requirements to detect a spatial feature at a desired power (dashed line) for a small (orange) versus large (green) FOV.

However, to the best of our knowledge, statistical frameworks tailored for power analysis for spatial profiling methods are currently lacking. Prior power analysis methods in genomics were devised in the context of either bulk profiling, in which the tissue is homogenized, or single-cell profiling^22–26^ (https://satijalab.org/howmanycells) in which cells are dissociated (Fig. 1a). In suspension experiments, there are a few relevant parameters for the sampling strategy: the overall number of cells and the relative abundance of different cell types (Fig. 1a). So far, spatial profiling studies have focused on detecting spatially resolved genes or specific cellular neighborhoods post hoc^27–30^, but have not considered questions of sampling strategy, such as the number of unique tissue samples or FOVs needed to reliably detect different patterns, or the effect of FOV size (Fig. 1b). Finally, power analyses that have previously been performed to address heterogeneity of single (bio)markers in whole tissues do not scale to novel profiling technologies^31^.

Spatial power analysis poses several challenges. First, spatial experiments offer a very large number of possible spatial features that might be relevant, and these features may be challenging to pre-define. Thus, in addition to distribution of cell-type proportions (as in single-cell genomics), cellular organization in the context of other cells and the tissue architecture are paramount, but such structures are difficult to parameterize and vary across tissues. Second, power analysis usually requires exploration of large amounts of data or a well-defined model of the system of interest to simulate the underlying distributions. In some settings (for example, addressing how FOV size impacts feature detection in one slide), it is possible to proceed directly from limited spatial data to power analysis (Fig. 1c), but other questions (for example, how many whole slide images are required to detect all significant cell–cell adjacencies in a cohort) require substantially more data, which may not be available.

Here, we introduce a power analysis framework to help design and interpret spatial profiling studies in tissues, including an approach to generate tissues in silico by parameterized models of tissue structure, overcoming limited data availability and serving as an approximate generative model for tissues. We illustrate spatial power analysis for several key questions for spatial biology, including the detection of cell types from spatial omics data, the detection of enriched cell–cell adjacencies, and the comparison of tissues and tissue cohorts on the basis of spatial organization. We show the impact of experimental design choices, such as the size, number, and placement of FOVs and spatial resolution, on the detection of spatial features, both when experiments are designed to detect known features and when the set of spatial features is unknown.

## Results

### In silico tissue framework for spatial power analysis

To begin to address the challenges of spatial power analysis, we generated tissues in silico by parameterized models of tissue structure (Fig. 1). We constructed blank tissue structures (‘tissue scaffolds’) and applied heuristic or optimization-based labeling solutions to generate in silico tissues (ISTs) that reflect parameterized spatial features and molecular information (Fig. 1d and Methods). To generate a tissue scaffold, which represents the spatial location of generic cells, we employ a random-circle-packing algorithm to generate a planar graph (Methods). Next, we assign an attribute labeling to the graph, where attributes on nodes represent cell type assignments. The labeling is based on two data-driven parameters for a given tissue type: the proportions of the *k* unique cell types, and the pairwise probabilities of each possible cell type pair being adjacent (a *k* × *k* matrix) (Fig. 1d, Supplementary Fig. 1 and Methods). We assume that these data-driven input parameters are available from prior knowledge or a pilot phase of a study. These parameters are local in nature and could vary across the tissue. For instance, tissues with known gross morphological regions may have different cell-type abundances and adjacency probabilities in each region. In such a case, using prior knowledge of the gross morphology, we generate sub-regions drawn from parameters corresponding to morphological regions, and stitch them to create a full IST (Fig. 1e and Methods). This generates a mosaic representation of tissue architecture. We then use this feature-independent framework to directly perform and validate power analysis results. Although we used cell-type labels as attributes, any type of attribute can be used.

### Spatial power analysis for cell-type detection

We first used ISTs for experimental design focused on cell-type detection in spatially resolved data, considering two sampling strategies: one in which single cells are observed in isolation from their spatial context, analogous to (non-spatial) single-cell profiling, and another in which spatially contiguous regions within tissue samples (‘sub-samples’ or ‘FOVs’) are observed (Fig. 1a,b). We constructed two statistical models to describe the corresponding probability of detecting a minimum number of cells of a particular (pre-defined) type in spatial sampling: a beta-binomial model to predict how many single cells need to be measured to observe a cell type of interest at a certain probability, and a gamma-Poisson model to predict how many FOVs are required to observe a cell type of interest at a certain probability (Methods). We then applied our framework to demonstrate how ISTs can be used to help experimental design for cell-type discovery in spatial profiling experiments. As a case study, we generated small ISTs with 2,186 cells, which approximates 500 × 500 μm in size, a typical size of one core in a tissue microarray (TMA)^1,5^. Next, we assigned one of four cell-type labels to cells in three spatial configurations: (1) a tissue in which a rare cell (3% abundance) is randomly located (Supplementary Fig. 2a, maroon); (2) a tissue with one cell type exhibiting strong self-preference (for example, that a given cell type is highly likely to be located adjacent to a cell of the same type) (Supplementary Fig. 2b, purple); and (3) unstructured tissues (serving as a null model) in which cells of all types have an equal probability of being adjacent to any other cell (given their proportions) (Supplementary Fig. 2c).

As expected, cell-type abundance greatly affected the number of cells and FOVs required to have a specified likelihood of observing a cell type of interest. For example, after sampling 20 cells in our null tissue, observing a common (abundance 22%) cell type of interest at least once was nearly guaranteed, whereas for a rare (3%) cell type of interest, sampling 100 cells gave just an 80% chance of detection (Supplementary Fig. 3a). Moreover, for ISTs with the rare-cell-type design, we asked how many FOVs of a fixed size (1%, 5%, or 10% of tissue area) are required for a given probability of observing the rare cell type in at least one FOV (Supplementary Fig. 3b). For example, at least three FOVs, each being 1% of the tissue size (~22 cells), must be examined to have an 80% chance of observing the rare cell type in at least one FOV (Supplementary Fig. 3b).

### Spatial power analysis for cell–cell adjacencies

We next used ISTs to determine the sampling strategy required to detect cell–cell adjacency patterns in a set of samples as compared with a null model. For this study, we use the term ‘cell–cell adjacencies’ to refer to direct adjacencies between cells in tissue (although the same framework can be used for other spatial proximities). To this end, we applied a permutation test^5,32^ to identify pairs of cell types that occur in proximity more (‘significant adjacencies’) or less (‘significant avoidances’) frequently than expected by chance (Methods), by comparing a real tissue to a null model. To simulate this setting, we generated two sets that each contained 25 ISTs (2,186 cells per IST)—one was structured by self-preference of one cell type (to simulate real tissue), and another followed a random tissue model (serving as the null)—and identified cell–cell adjacencies that characterized structured ISTs compared with the random (null) tissue model (permutation test *P* < 0.01, Methods). Hierarchical clustering of the permutation-test results showed that the self-preference ISTs consistently had the desired adjacency, but the randomly structured set did not (Supplementary Fig. 4a).

To simulate a more complex structure, we generated another set of 25 structured ISTs with an enriched adjacency between three of ten cell types, and 25 random ISTs with the same ten cell types but without any constraints on the adjacencies. Again, hierarchical clustering of the permutation-test scores for each pair of cell types separated structured ISTs from non-structured ISTs, with the enriched adjacency recovered in only the structured set (Supplementary Fig. 4b). Next, we showed how tissue sets could be separated on the basis of adjacencies, by testing whether the distributions of significance scores for each adjacency were significantly different between the structured and unstructured ISTs for different numbers of tissues. We found that the specified adjacencies were among those with distinguishable score distribution, even when only a small number of tissues was compared (Supplementary Fig. 4c).

### ISTs can recapitulate real tissues of different structures

**Fig. 2:**
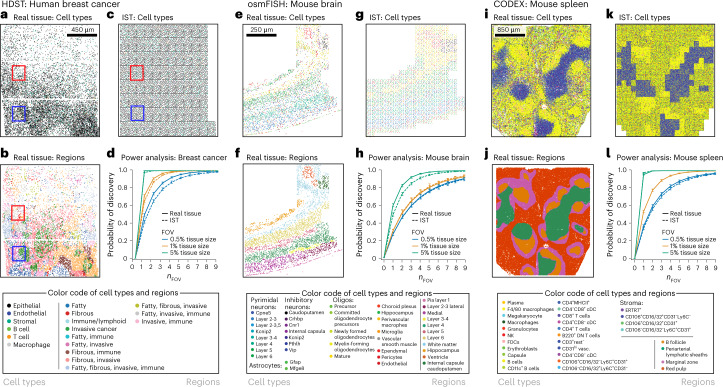
Spatial power analysis to recover cells and cellular adjacencies of interest using ISTs in different tissue types. **a**–**l**, Power analysis of the number and size of FOVs required to detect rare cell types by spatial analysis of tissues with different structures. **a**–**d**, HDST data set of breast cancer. **a**–**c**, Cells (points) at their spatial position in real (**a**,**b**) and corresponding IST (**c**) data, labeled by type (**a**) or morphological regions (**b**). Areas within the red and blue rectangles are expanded in Supplementary Figure 5. **d**, Probability (*y* axis) of discovering at least one T cell when sampling different numbers of FOVs (*n*_FOV_, *x* axis) of different sizes (colored lines), in either real tissues (solid lines) or ISTs (dashed lines). **e**–**h**, osmFISH murine cortex data. **e–g**, Cells (points) at their spatial position in real (**e**,**f**) and corresponding IST (**g**) data, labeled by type (**e**) or morphological region (**f**). **h**, Probability (*y* axis) of discovering at least one L6 pyramidal neuron when sampling different numbers of FOVs (*x* axis) of different sizes (colored lines), in either real tissues (solid lines) or ISTs (dashed lines). **i**–**l**, CODEX mouse spleen data. **i**–**k**, Cells (points) at their spatial position in real (**e**,**f**) and corresponding IST (**g**) data, labeled by type as defined by protein expression (**i**) or morphological region (**j**). **l**, Probability (*y* axis) of discovering at least one megakaryocyte when sampling different numbers of FOVs (*x* axis) of different sizes (colored lines), in either real tissues (solid lines) or ISTs (dashed lines). **d**,**h**,**l**, Error bars indicate a 95% confidence interval calculated over 50 independent experiments (30 independent FOVs drawn at each size per experiment).

Next, we applied our approach to parameters derived from three real biological data sets: a high-density spatial transcriptomics (HDST) data set of breast cancer, a cyclic-ouroboros single-molecule fluorescence in situ hybridization (osmFISH) data set of the mouse cortex, and a highly multiplexed antibody-based (CODEX) murine spleen data set^11,14,17^ (Supplementary Table 1). In HDST, microwells are spatially barcoded with 2-μm beads that enable transcriptome-wide RNA capture; in osmFISH, 33 cell-type-specific RNA markers are targeted through a cyclic single-molecule fluorescence in situ hybridization process; and in CODEX, antibody–antigen binding events are visualized through sequential decoding of DNA barcodes uniquely coupled to a 30-plex antibody cell-type-specific panel. In each case, we used available gross morphological data to estimate cell-type abundance and pairwise adjacency probabilities from each annotated morphological region in the data set and generated ISTs on a tile-by-tile basis using region-specific estimates of spatial parameters and our heuristic labeling strategy to speed computation (Fig. 2a–l and Methods).

These data sets span a broad range of complexity of biological structure. The HDST breast-cancer data are relatively unstructured; despite provided annotations of morphological zones, the tissue is dominated by one cell type (epithelial cells) with little variation in composition between morphological zones (Fig. 2a,b and Supplementary Fig. 5). The mouse cortex is a highly ordered, layered tissue with unique cell types in each morphological zone (Fig. 2e,f), and the mouse spleen has complex, recurrent structure with shared features between morphological zones of the same type (Fig. 2i,j).

### Tissue structure impacts power for cell-type detection

Power analysis shows how the extent and nature of tissue structure impacts the number of cells and FOVs required for cell-type detection. In each of three real data sets, we selected a cell type of low abundance to better illustrate the effect of sampling strategy on feature recovery (as highly abundant cell types would be detected universally). We implemented three sampling strategies: (1) sampling FOVs and assaying them in their entirety (‘spatial sampling’) for the presence of a cell type of interest (for example, analysis of a TMA or a specified ROI; Fig. 2d,h,l); (2) sampling FOVs, dissociating each, and profiling a certain number of cells in each FOV (‘regional sampling’, Supplementary Fig. 6); and (3) dissociated single-cell analysis of the entire tissue sample, such that no spatial information is retained (‘single-cell sampling’, for example flow cytometry or single-cell RNA sequencing; Supplementary Fig. 6b,d,f, dash-dotted lines (Random)). In each cell-discovery experiment, we varied one experimental design parameter (for example, number of FOVs or FOV size) while holding all others constant (Fig. 2). For spatial sampling, we used a gamma-Poisson model to determine the number of FOVs of a fixed tissue area that is required to detect at least one cell of a cell type of interest in real tissue or in its corresponding IST (Fig. 2d,h,l). We used fixed tissue areas, because FOVs may have varying cell counts owing to different cell densities, which is accommodated by the model. For regional sampling (for example, when FOVs are drawn then dissociated), after FOVs are sampled, we used a beta-binomial model to estimate the number of cells captured from each FOV (Supplementary Fig. 6b,d,f; solid and dashed lines). For non-spatial single-cell sampling, which does not capture any spatial information and is equivalent to a FOV sized to capture one cell, we employed a binomial process with the same assumptions. We assumed that a cell type is completely determined by its markers and defined detection as observing at least one cell of the type in a FOV; however, our model can accommodate any desired threshold of detection. In each case, we can model the effect of cell-type miscalling by adjusting the estimated abundance of a cell type of interest with a false discovery rate (this effectively makes the cell type rarer and will thus increase sampling requirements).

With spatial sampling, in the relatively unstructured breast cancer tissue, there is an 80% probability of detecting a T cell in one FOV that is 5% (~500 cells) of the total tissue size (Fig. 2d). In the mouse cortex, where the tissue is highly structured and non-repetitive, attaining an 80% probability of detecting one of the more abundant L6 pyramidal neurons (9% abundance) (Supplementary Fig. 6c) requires two FOVs that are each 5% (~650 cells total) of the tissue area (Fig. 2h). Finally, in the mouse spleen, the repeated morphological structures (for example, periarterial lymphatic sheaths and B follicles surrounded by a marginal zone) lower the number of FOVs that is required to detect even very rare cell types, such as megakaryocytes (~0.1% abundance): just one FOV (~4,300 cells) that is 5% of the tissue area is sufficient to confer a probability of >80% of detecting at least one such cell (Fig. 2i–l). There is also an 80% probability of detecting a megakaryocyte by sampling four FOVs, each being 0.5% of the tissue area (~1,700 cells total), illustrating the impact that sampling strategy has on the absolute number of cells required to detect a spatially distributed feature. With non-spatial single-cell sampling, by contrast, profiling ~100 cells in breast cancer tissue would achieve an 80% probability of detecting at least one T cell (Supplementary Fig. 6a,b); 17 cells suffice to detect at least one L6 pyramidal neuron at 80% probability in the mouse cortex (Supplementary Fig. 6c,d); and ~1,270 cells are required to attain an 80% probability of detecting a rare megakaryocyte in the mouse spleen (Supplementary Fig. 6e,f). Thus, power analysis considering only overall cell frequencies would vastly underestimate the number of FOVs that is required for a spatial experiment.

Smaller FOVs were less impacted by spatial overdispersion, where the observed variance in a data set is higher than expected. In the hypothetical limit of a FOV sized so small that it can capture only one cell, there is no spatial overdispersion by definition, and this situation is statistically equivalent to single-cell sampling. When a cell type is spatially overdispersed in the context of a highly ordered and heterogeneous tissue (mouse cortex), multiple smaller FOVs yield better detection probability than a single larger FOV (Fig. 2h), but this is not the case in tissues with more repetitive organization (spleen). Sampling experiments on both real data and their corresponding ISTs generally agreed, suggesting that our ISTs can recapitulate tissue properties for this purpose. Although we normalized FOV size relative to the total area of the tissue sample, absolute tissue size is important because biological features exist at different length scales (for example, a FOV or another spatial sub-sample that is entirely within a tissue sub-region that lacks a certain cell type will never result in the discovery of that specific cell type).

### Power analysis for detection of cell–cell adjacencies

Next, we used our framework to detect significant cell–cell adjacencies in real data. We defined significant adjacencies and avoidances through a permutation test^5,32^, as described above (Methods), determined the number of FOVs required to detect any significant finding, and estimated how selection of FOV size impacts the types of detectable adjacencies. Focusing on spleen as a case study, we examined CD4^+^ and CD8^+^ T cell adjacencies, which are enriched in the full tissue (*P* < 0.01, permutation test, Methods). Using our IST, we estimate that measuring >7.5% of the assayed tissue size (~123 × 123 µm, ~5,600 cells) would recover this adjacency as significant (permutation test, *P* < 0.01) at 80% probability, with a sharp inflection point (Supplementary Fig. 7a,b). This inflection point reflects the FOV size at which spatial sub-samples are being drawn on the length scale of macroscale spatial organization, and should be accounted for when sampling with fixed FOV sizes, as in the case of TMAs. TMAs of insufficient size may never capture the feature of interest. In general, areas in which the adjacency of interest is recovered span across morphological zones, such that they are representative of the diversity of tissue structures (Supplementary Fig. 7c, green squares).

### Detecting differential adjacencies between tissues or cohorts

**Fig. 3:**
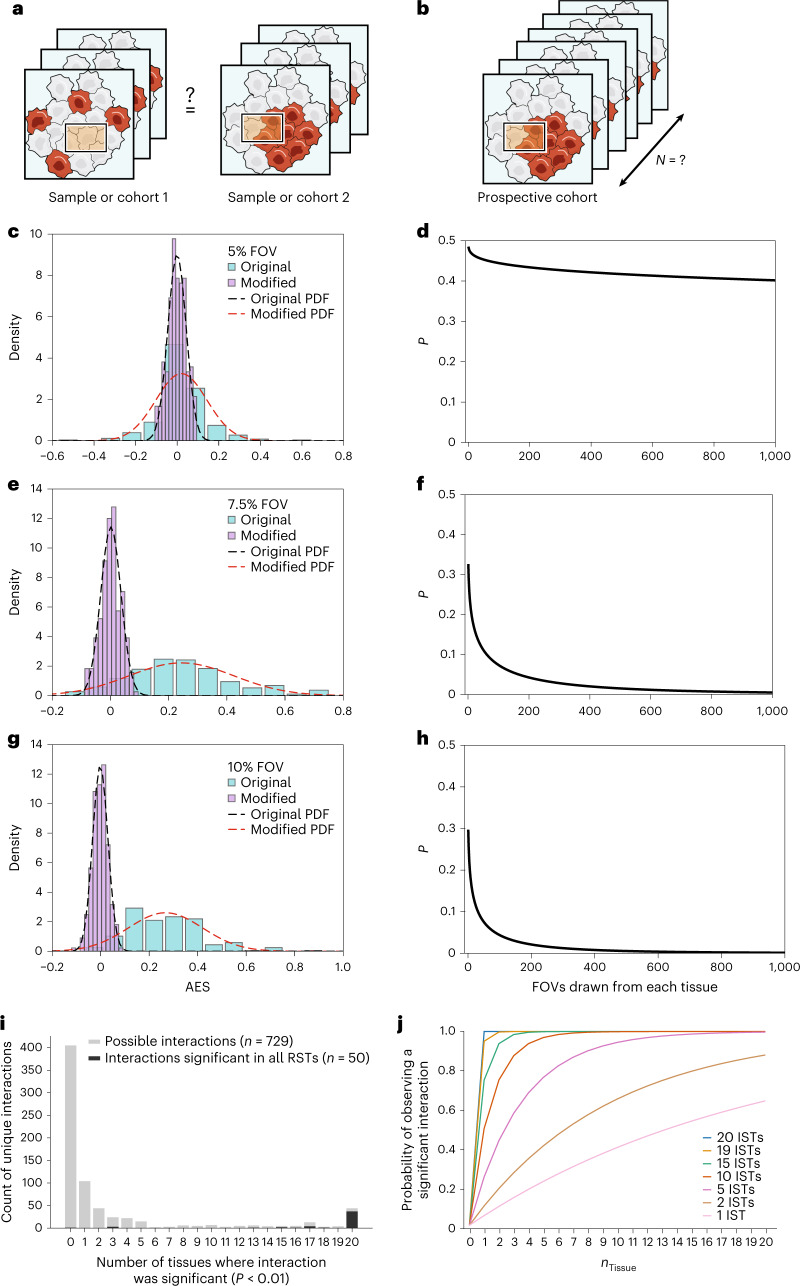
Comparing tissue cohorts on the basis of spatial structure. **a**,**b**, Spatial power analysis provides insight into experimental design. Schematic of design to distinguish cohorts (or individual samples) (**a**) or select cohort size to detect a spatial feature of interest (**b**). **c**,**e**,**g**, Larger FOVs distinguish tissues with different AESs. Distribution of AESs for FOVs of 5% (**c**), 7.5% (**e**), or 10% (**g**) of the absolute tissue size, drawn from the original spleen tissue (cyan) or a modified data set in which the overall number of CD4^+^–CD8^+^ T cell adjacencies was reduced by 37% but the relative frequency of cell types and absolute structure were preserved (magenta). Dashed lines, probability density functions for the original (red) and modified (black) tissues. **d**,**f**,**h**, Power analysis for comparison of distributions of AES between original and modified tissues. *P* value (*y* axis) in a *t*-test comparing the AES distribution of original and modified tissues (as in **c**,**e**,**g**), for different numbers of FOVs (*x* axis) that were 5% (**d**), 7.5% (**f**), or 10% (**h**) of the absolute tissue size. **i**,**j**, Power analysis for number of tissues required to detect a significant cell–cell adjacency. **i**, Distributions of number of unique cell–cell adjacencies (*y* axis) detected as significant (*P* < 0.01, permutation test, one-sided) in a set of 20 ISTs (*x* axis), for 729 possible cell–cell adjacencies (light gray) and for the 50 cell–cell adjacencies that were significant in all three real spleen tissues (dark gray). **j**, Probability of observing a significant adjacency (*y* axis) for a different number of tissues sampled (*x* axis) for adjacencies recovered in different numbers of ISTs (line color).

In certain experimental designs, researchers may ask whether there is a difference in the significance of an adjacency between two tissues (or two cohorts of tissues) and assess sampling requirements to achieve statistical power to detect differentially significant adjacencies (Fig. 3a) or to predict the necessary cohort size to detect a spatial feature of interest (Fig. 3b). To assess this, we constructed the adjacency enrichment statistic (AES), which quantifies the enrichment of a specific cell–cell adjacency, defined here as the frequency of a specific adjacency relative to the frequency expected given the proportion of the two cell types (Methods). By relying on the expected frequency, we can rapidly compute the AES without permutations and then compare the two tissues (Methods).

We tested this approach in the context of CD4^+^ and CD8^+^ T cell adjacencies in the spleen. We analyzed the real mouse spleen data set along with a copy in which we had rearranged cells in adjacent CD4^+^–CD8^+^ pairs to reduce the frequency of the CD4^+^–CD8^+^ T cell adjacency by 37% while preserving the overall cell-type frequency and tissue structure (Methods). We then drew 100 FOVs of fixed size (5%, 7.5%, or 10% of full tissue size) from each of the two tissues and calculated the AES for the CD4^+^–CD8^+^ T cell adjacency in each FOV, yielding an AES distribution. Finally, using the maximum likelihood estimate of the mean and variance, we fit a Gaussian to the AES distribution.

FOV size has a substantial impact on the ability to detect differentially significant adjacencies between tissues (Fig. 3c–h). With a 5% FOV, the CD4^+^ and CD8^+^ T cell adjacency is only rarely significantly detected (Fig. 3c), and we cannot identify it as differentially significant between the two tissues (*P* = 0.41, *Z*-test). Systematically testing how FOV size and effect size affect power in this setup (Fig. 3b), we found that, when the FOV size is increased, the differentially significant adjacency is readily detected (7.5% FOV, *Z*-test *P* = 0.018; 10% FOV, *Z*-test *P* ≪ 0.01, Fig. 3e–h). Thus, a smaller number of FOVs (sub-samples) is required to distinguish the difference in adjacency enrichment by the AES test (*P* = 0.05) as the FOV size grows (~1,000, ~100, and ~50, respectively). Because AES measures enrichment relative to the proportions of the cell types that are present in a sample, this analysis assumes that these proportions are equal between samples.

### Sampling requirements for unknown features

We next showed how our in silico framework can be used to make predictions of sampling requirements when the set of true features of interest is unknown (in contrast to pre-specified cells or adjacencies above). To this end, we assembled a set of three real mouse spleen tissues, estimated the input parameters for IST generation from one of these three tissues, and held the remaining two for validation. We generated 20 ISTs on the basis of the estimated parameters; this number was selected to capture a broad set of cell–cell adjacencies that can be spuriously detected as significant given the input parameters or biological noise (in real data). Unlike in previous analyses, we aimed to enumerate a set of statistically significant spatial features, rather than to recover a known ground truth. Given this goal, and the fact that our tissue-generation approach does not recapitulate macrostructures natively, there is a risk that repeating macrostructure layout in all ISTs could generate spurious adjacencies. To address this, we shuffled macrostructures on the basis of regional annotations included in the real data set (Methods), and then called significant cell–cell adjacencies in the ISTs individually and in the data set overall (Permutation test, *P* < 0.01).

Of 729 possible pairwise adjacencies, only 69 were significant in more than 80% of ISTs, of which 44 were significantly enriched in all 20 ISTs (Fig. 3i, gray). Importantly, of the 50 adjacencies that were significant in all three real tissues, 37 (84%) overlapped with the 44 that were significant in all 20 ISTs (Fig. 3i, black). Another 13 were identified as significant in real spleen data, but were not among the 44 adjacencies that were detected as significant in all ISTs and were largely associated with cell types at the boundaries in the segmentation mask or tissue (Supplementary Fig. 8a,b). To predict the number of tissue samples that is required to observe a specific adjacency at a desired probability, we calculated the proportion of ISTs in the set in which we observed a specific adjacency (Fig. 3j). For example, to detect at 80% probability an adjacency of interest that occurs in just 5 of 20 ISTs, an experiment should have at least six tissue samples.

### Lower spatial resolution increases sampling requirements

Finally, we examined the impact of spatial resolution on sampling requirements. Unlike the HDST, osmFISH, and CODEX data sets, which all have single-cell or near-single-cell spatial resolution, other popular methods, such as Spatial Transcriptomics^12^ (commercially available as Visium), currently enable transcriptome-wide mapping of spatial domains at 55-μm resolution^12^ (https://www.10xgenomics.com/products/spatial-gene-expression). To test the impact of spatial resolution on sampling requirements, we spatially binned the CODEX spleen and HDST breast cancer data sets to a resolution comparable to that of Visium (55 μm; Methods), and repeated our power analysis for cell-type detection, asking how many FOVs of a fixed tissue size were required to detect at least one cell of the cell type of interest after cell-type deconvolution (Methods).

In both spleen and breast cancer tissues, at Visium-like resolution, a larger number of FOVs was required to detect a given cell type at the same probability than in the higher resolution assays. For example, seven FOVs that are each 1% of the tissue area are required for an 80% probability of detecting a given cell type in the spleen with Visium-like data (Supplementary Fig. 9a), compared with three FOVs that are each 1% of the tissue area with CODEX data (Fig. 2l). Sampling FOVs from multiple CODEX tissue samples did not have a benefit compared with sampling more FOVs from the same tissue sample, with a similar penalty for data of lower spatial resolution (Supplementary Fig. 9b). Similarly, in breast cancer tissue, Visium-like data required five FOVs, each being 1% of the tissue area, to detect at least one T cell (Supplementary Fig. 9c), compared with only two FOVs with HDST (Fig. 2d), and there was surprisingly no benefit for multiple distinct tissue samples versus a single sample (Supplementary Fig. 9d).

## Discussion

In this study, we developed an in silico tissue framework to enable spatial power analysis and assist with experimental design. In addition to their use in experimental design, ISTs can be directly used for method development and benchmarking of existing^29,30,32,33^ or novel spatial analysis methods^34^.

In our framework, we used cell-type labels instead of individual quantitative features (for example, marker intensity or cellular morphology) to provide a straightforward and interpretable abstraction, but any spatial profiling data can be used. In all cases, our power analysis based on individual ISTs accurately predicted the probability of cell detection compared with the real tissue, showing that IST generation mimics actual tissue structure given estimated parameters from a variety of spatial profiling data types and underlying tissue structures (Fig. 2a–l). However, in the case of the HDST breast cancer data set, we observed some deviation between predictions of sampling requirements using our approach on real data and on ISTs; we believe that this can be accounted for by the fact that this data set contains uneven densities of cells, which we did not explicitly model. Future work could extend our approach to include this consideration to improve IST generation for tissue types with highly variable cell densities. Overall, we robustly created ISTs across diverse tissue types and various experimental methods to perform accurate spatial power analysis for cell-type detection. Additionally, although both the heuristic and optimization-based labeling methods can achieve similar results, our formulation of the optimization problem is related to *k*-graph coloring problems, some formulations of which are known to be NP (nondeterministic polynomial time)-complete. Thus, optimization can prove difficult and computationally slow in practice, in which case the heuristic labeling strategy is a useful substitute (Methods).

Although retrospective power analyses could be performed on sufficiently large extant biological data sets, this is not necessarily practical for designing new spatial experiments in which the particulars of spatial structure impact power. As an alternative, ISTs enable predictive spatial power analysis to inform experimental design decisions early in a study, depending on the feature of interest. We provide a tool to create ISTs, perform statistical testing to identify spatial features, simulate different experimental design choices, and perform spatial power analysis.

Using this framework, we enumerated some parameters for consideration in the design of spatial experiments, including tissue size, diversity of cell types, spatial structure, sampling strategy (for example, TMA size selection), and feature of interest (for example, cell-type discovery or spatial motif discovery). Additionally, we applied our approach to examine several experimental design questions unique to spatial omics. For example, we showed that sampling requirements (for example, the number of FOVs) are contingent on the spatial technology used, such that experimental methods with lower spatial resolution increase sampling requirements compared with methods with single-cell resolution. Although we chose to abstract our analysis to the detection of cell types and spatial relationships between cell types, our work also sheds light on the impact of error-prone cell-type calling and cell-type deconvolution on sampling requirements. Finally, we begin to examine whether sampling the FOVs from multiple tissue samples confers a statistical advantage over sampling multiple FOVs from one tissue sample. In our specific analysis, we find that there is no significant benefit, suggesting that there is only narrow spatial heterogeneity between individual tissue samples in some settings. However, our analysis is limited by the data sets used in this study, which contain relatively few tissue samples collected at near-adjacent distances from the biopsies. A complete assessment of the statistical nature of biological variability between different samples and its impact on sampling requirements to distinguish cohorts of people using spatial omics data would require a more thorough analysis of larger cohorts. Substantial and important work remains to better elucidate biological variability of both tissue macrostructure and person-to-person variation to provide a robust answer to this question. In summary, our work will aid researchers in designing more statistically principled experiments for extracting meaningful biological or clinical insights from spatial omics studies.

## Methods

### In silico tissue generation

In silico tissues were generated by first constructing a tissue scaffold—a blank tissue with no cell information assigned—then assigning cell-type labels to the scaffold.

### Generating tissue scaffold

Tissue scaffolds were generated with a random-circle-packing algorithm. This algorithm places circles of a bounded random radius within a rectangular region, disallowing overlaps between circles through rejection sampling. The algorithm continues until it fails to place any new circle 500 consecutive times. This results in a densely packed region, though density can be tuned by adjusting the allowable circle radii. In this model, circles represent cells. Touching circles represent adjacent cells and will be connected by an edge in the graph representation (Fig. 1d).

Circle-packing results are then converted into a graph representation. A graph is a highly interpretable data structure that can represent a tissue owing to its clear encoding of spatial relationships and ability to be labeled with biological information. This is performed by calculating, for each circle, all other circles within the smallest allowable radius of the original circle’s perimeter. Effectively, for a circle *C*, this finds all circles that *C* would overlap with if the radius of *C*, *r*_*c*_, was modified such that *r*_*c*_ = *r*_*c*_ + *r*_min_, where *r*_min_ is the smallest radius. These circles are considered to be adjacent to *C*. A node is placed at the center of each circle, and an undirected edge is drawn to the node corresponding to each of the adjacent circles (Fig. 1d). These graphs were implemented using NetworkX 2.6.2.

### Assigning cellular information

After generation of the tissue scaffold, cellular information was assigned to the tissue. Two input parameters were specified in this process, a vector $p \in {\Bbb R}^K$, which contains the probabilities of discovering each of the *K* cell types in the tissue. Further, a matrix $H \in {\Bbb R}^{K \times K}$ is defined where $h_{ij} \in H$ defines the probability that a cell of type *k*_*i*_ is adjacent to a cell of type *k*_*j*_. Two alternative algorithms were used to assign labels to the tissue scaffold.

### Graph neighborhoods and heuristic assignment

A neighborhood, *N*_*v*_, was defined on the graph, *G*, representation of the tissue scaffold. For a vertex $v \in {\it{G}}$, *N*_*v*_ = *G*[*S*] is defined as the subgraph induced by the set $S = \left\{ {u \in G|d\left( {v,u} \right) \le {\it{\epsilon }}} \right\}$, where *d* is a function computing the geodesic distance, *u* and *v* are nodes in *G*, and *∈* specifies the search radius.

The graph region was partitioned into a grid of regions of 50 × 50 px. Within each region, a start node (*v*_*i*_) was selected at random. The type ($k_{v_i}$) of *v*_*i*_ was sampled from a multinomial distribution of the cell type probabilities: $K_{v_i} \sim Multinomial\left(\,p \right)$, where *p* is the cell type distribution. Given the choice of *k*, the probabilities of the type labels for the nodes $v_n \in N_{v_i}$ are sampled from a multinomial distribution of the corresponding row vector in *H*, $v_n \sim Multinomial\left( {H_{k \ast }} \right)$.

The partition grid is then shifted horizontally and vertically by 25 px, and the sampling process is repeated. Any remaining unlabeled nodes are then discovered and assigned by the same process. After all nodes are labeled, random nodes are selected, and the observed neighborhood label distribution (${\hat{H}}$) is calculated and compared with $H_{k \ast }$. Overabundant type labels in ${\hat{H}}$ are swapped to under-abundant type labels. Typically, a well-behaved tissue, which reasonably approximates the initial specifications, can be constructed in a few hundred iterations.

### Optimization of cell assignment

#### Setup

Given the blank tissue scaffold, an assignment matrix $B \in {\Bbb R}^{n \times K}$ is computed that describes the cell-type assignment for *n* cells in the tissue scaffold. An entry *B*_*vk*_ = 1 if a node *v* is of type *k*, otherwise *B*_*vk*_ = 0. Furthermore, as each cell may receive only one type assignment, each row in *B* sums to 1, $\mathop {\sum}\nolimits_{j = K}^K {b_{ji} = 1}$ and each column sums to the expected cell-type count, which, when normalized, yields the cell type distribution (*p*). In a fully labeled tissue with adjacency matrix *A*, the matrix of neighborhood probabilities *H* given an assignment *B* can be computed as:

1 $$H\left( B \right) = diag\left( {B^ \top B} \right)^{ - 1}B^ \top diag\left( {AA^ \top } \right)^{ - 1}AB,$$

where $diag\left( {B^{\it{ \top }}B} \right)$ is the count of each cell type, $diag\left( {AA^{\it{ \top }}} \right)$ is the number of edges per node in the graph, and *AB* is the number of neighbors by type (columns) per node (rows). Combining these terms as described in Equation 1 yields a *K*×*K* matrix in which an entry (*i, j)* is the proportion of neighbors of cells of type *i* that are of type *j*.

#### Objective

Given a target matrix of neighborhood probabilities $H\left( {B^ \ast } \right)$ derived from real data and a random synthetic tissue scaffold with its resulting adjacency matrix *A*, probabilistic synthetic assignments of cells to labels are generated that conserve observed neighborhoods of cell label to cell label preferences.

This problem is formulated as an inverse optimization problem, in which we seek to find a probabilistic assignment matrix $B \in {\Bbb R}^{n \times K}$ that would lead to a matrix of neighborhood probabilities *H*(*B*) matching the observed data as closely as possible.

The resulting objective aims to recover a matrix *B* representing synthetic data that optimizes the loss:

2 $$\begin{array}{l}argmin_B\left\| {H\left( B \right) - H\left( {B^ \ast } \right)} \right\|_2,\\ \text{such that}\mathop {\sum}\limits_k {B_{ik} = 1,\,B_{ik} \in \left\{ {0,1} \right\},B\,1_K = 1_n,} \\ 1_n^ \top B = np,\end{array}$$

where, as before, *p* is the cell type distribution we aim to match, n is the number of cells, *k* is a generic placeholder for the cell types encoded in *K* and 1_*K*_ represents a *K* dimensional vector of ones. When the assignment is required to be unique and all the entries of *B* are integers, the question of whether such a labeling exists is generally difficult to settle. In a particular case, if cells sharing the same label exhibit strong repulsive behavior towards one another such that the neighborhood probabilities $H\left( {B^ \ast } \right)$ is a matrix with zero diagonal, without the constraint $1_n^{\it{ \top }}B = np$, the optimization problem is akin to the well-known vertex graph coloring problem^35^. In the *k*-coloring vertex problem, the aim is to decide whether a graph can be colored using *k* colors such that no vertices of the same color share an edge. For *k* > 2, this problem and many of its variants are known to be NP-complete.

The considered loss is further equivalent to the semidefinite program objective:

3 $$\begin{array}{l}argmin_B - Trace\left( {H\left( B \right)^TH\left( {B^ \ast } \right)} \right)\\ \text{such that} \mathop {\sum}\limits_k {B_{ik} = 1,\,B_{ik} \in \left\{ {0,1} \right\},B\,1_K = 1_n,} \\ 1_n^ \top B = np\end{array}$$

Finally, an efficient algorithm is derived to solve a relaxed version of this problem by considering the augmented Lagrangian objective over a matrix B with continuous entries:

4 $$argmin_B - Trace\left( {H\left( B \right)^TH\left( {B^ \ast } \right)} \right) + l_1\left\| {B\,1_K - 1_n} \right\|_2 + l_2\left\| {1_n^TB - p} \right\|_2,$$

for positive real parameters *l*_1_ and *l*_2_.

### Implementation details

For GPU accelerated automatic differentiation, the optimization routine was implemented using JAX 0.2.27 in Python 3.7.11 (www.github.com/google/jax). Further details regarding implementation, system requirements, and demo instructions are available at https://github.com/klarman-cell-observatory/PowerAnalysisForSpatialOmics. For details regarding optimizing the augmented Lagrangian objective, see the function optimize_assignment in the spatialpower.tissue_generation.assign_labels module.

### Parameter optimization

The expected cell-type proportion, *p*, and the expected neighborhood distribution matrix, *H*, are directly provided by the user. The optimization routine has additional parameters. The learning rate and two additional loss weight parameters, ‘l1’ and ‘l2’ are set. The two parameters ‘l1’ and ‘l2’ weigh the relative contribution of constraints on the bounds of the probabilistic assignment and *p*, respectively. In detail, the first parameter enforces that all the *n* rows of *B* sum to one; the second one enforces that the resulting solution *B* marginally matches cell-type proportions (columns sum to the desired expected numbers of cells of a given cell type).

Note that, in its current form, the objective enforces (through the term dominated by ‘l2’) that the assignment *B* matches the cell-type proportions uniformly. Since the constraint parameters are additive, the objective can be encouraged to be more biased toward populations of cell labels that, owing to their rarity, might otherwise be overlooked. This is accomplished by introducing optional, cell-label-specific parameters (*w*_*k*_) to control the relative contribution of the specific constraints on *p*, invoking a tradeoff between unique assignment and matching assignments to *p*. The corresponding objective is:

5 $$argmin_B - Trace\left( {H\left( B \right)^TH\left( {B^ \ast } \right)} \right) + l_1\left\| {B\,1_K - 1_n} \right\|_2 + l_2\mathop {\sum }\limits_{k = 1}^K w_k\left( {1_n^TB\,e_k - p_k} \right)^2,$$

where *e*_*k*_ is the standard basis vector of dimension *K* with non-zero value at index *k*. For example, when dealing with a rare cell type—low *p*_*k*_—a higher weight (*w*_*k*_) will enforce that the rare cell type is going to have a non-zero chance of appearing in the resulting synthetic cell assignment.

### Incorporating cell-type proportion information

Owing to inherent tradeoffs between optimizing with respect to *p* and *H* jointly (the objective is sufficiently close to a graph coloring problem that ideal solutions may not be possible), it is desirable to assert control over which specific adjacencies are favored in the optimization process. Because a user may have prior knowledge about which adjacencies are the most or least abundant (‘extreme values’), an option is provided to optimize only over those elements of *P* that are beyond one s.d. from the mean. This favors extreme values in *P* by changing which values in *w*_*k*_ the *l*_2_ constraint is applied to (see ‘extreme_values’ in ‘constraint()’ in the tissue_generation.assign_labels.optimize module).

### Cell-type heterogeneity

The same general procedure is used to perform all sampling experiments, but with different models depending on the specific power analysis question at hand. Overall, the steps in this procedure are as follows: (1) obtain pilot data (or, potentially, literature estimates), (2) use pilot data to estimate parameters of the sampling model, and (3) use the model to make predictions of sampling requirements for a spatial experiment. Models return a probability of detecting a cell of a specified type given some level of sampling (for example, number of cells or FOVs).

To model the number of cells that must be measured to achieve a desired probability of observing a given number of cells of a specific type in a single-cell profiling experiment, the proportion of cells in a tissue that were of the type of interest is calculated. A simple binomial model is used to predict the number of cells that need to be profiled to achieve a certain probability of observing the cell type of interest *N* times. In the cell type detection experiments, we set *N* = 1.

Owing to underlying tissue structure that results in overdispersion in the number of cells of each type per FOV, the number of FOVs with a certain number of observed cells of a particular type is modeled by a gamma-Poisson (negative binomial) model. The negative binomial distribution (NBD) was used with the following parameterization:

6 $$P\left( {X = x} \right) = \frac{{\Gamma \left( {k + x} \right)}}{{x!\Gamma \left( k \right)}}\left( {1 + \frac{m}{k}} \right)^{ - k}\left( {\frac{m}{{m + k}}} \right)^x$$

for $x \in Z^{\,0 + }$ and where *m* > 0 and *k* > 0 are parameters describing the mean and shape, respectively. Estimated NBD parameters by moments estimation and the zero term method (ZTM)^36^ were compared. Owing to the high frequency of FOVs with no cells of the type of interest, the ZTM estimator achieved superior performance. We estimated:

7 $$\widehat m = \frac{1}{n}\mathop {\sum }\limits_{i = 1}^N x_i$$

To estimate ${\hat{k}}$, we numerically solve the equation:

8 $$\frac{{n_0}}{N} = \left( {\frac{m}{k} + 1} \right)^{ - k}$$

where *N* is the sample size and *n*_0_ is the count of zeros. The numerical solution was computed with the ‘fsolve’ function in SciPy 1.6.2 (ref. ^37^). Additional computations were performed using NumPy 1.20.3. A probability of discovery was computed by computing the complement of the model evaluated at the zero count, but the NBD describes the probability of describing any number of cells of the type in the FOV. Furthermore, the NBD can accommodate the fact that FOVs vary in the number of cells they contain (for example, because of differences in cell density across tissues). Importantly, this model also assumes that a specific combination of makers has 100% accuracy to define the cell-type label.

Finally, the number of cells required to be obtained via a FOV sampling strategy (‘regional sampling’) was modeled with a beta-binomial model, which, like the gamma-Poisson model above, accounts for spatial overdispersion owing to the underlying spatial distribution of cells of each type. The parameters of the beta-binomial distribution $\hat \alpha = \mu \left( {\frac{{\mu \left( {1 - \mu } \right)}}{\sigma } - 1} \right)$ and $\hat \beta = \left( {1 - \mu } \right)\left( {\frac{{\mu \left( {1 - \mu } \right)}}{\sigma } - 1} \right)$ were estimated, where *μ* and *σ* are the sample mean and variance, respectively. To calculate *μ* and *σ*, 50 randomly placed FOVs of 5% tissue area were drawn and the numbers of cells of the type of interest contained within each FOV were counted. The probability of detecting at least *N* cells of the type of interest at a given sampling level (for example, the number of cells sampled) is calculated using the beta-binomial survival function evaluated at *N* – 1 (because the survival function is defined with an exclusive lower bound).

### Full image creation

To construct ISTs at the scale of whole slide images, the image was compartmentalized into distinct morphological regions representing unique macrostructures (Fig. 2). All data sets selected for this analysis contained domain expert macrostructural annotations. For each, the required parameters for tissue generation were estimated from all annotated macrostructural zones. A lower-resolution segmentation map was generated by partitioning the original segmentation map into a grid and determining the dominant zone in each grid partition. Small tissue scaffolds were generated on the basis of the mean number of cells per grid partition and one assignment solution per grid square (tile) was generated. The parameters used in each tile matched the dominant zone in that tile. Tiles were stitched together to generate a composite image (Fig. 2). To save computational time in large images, only one blank tissue scaffold was generated and then relabeled for each tile. This approach additionally enables simpler stitching of tiles, although it does create an artifact during visualization because of a high density of points on the boundaries. Because our model considers only graph connectivity, this is a drawback only during visualization.

### Visualization

For small ISTs, a tissue-like representation was generated by computing a Voronoi diagram and coloring each Voronoi region with a color representing the cell type assignments (Supplementary Fig. 2). For larger ISTs, computing the Voronoi diagram can be slow. In this case, the tissue is visualized as a scatter plot, colored by cell type assignment.

### Tile shuffling

To determine the effects of tissue macrostructure in the murine spleen data, 20 full-size ISTs were generated with randomized macrostructure. Each of these ISTs contained the number of tiles from each zone as found in the original segmentation map. Tiles were generated as described above, but randomly stitched to generate shuffled images.

### Neighborhood discovery via permutation testing

Tissues with a significant pairwise cell-type adjacency, identified as pairs of cell types that are adjacent to each other more (‘significant adjacencies’) or less (‘avoidances’) frequently than expected were generated via a permutation test^5,32^, implemented as previously described^32^. In the permutation test, the ground truth neighborhood distribution matrix, *H*, was calculated, as described in equation (1). Then, the assignment labels on the tissue are shuffled to relabel the tissue, preserving the tissue structure. At each shuffle, the observed neighborhood distribution (${\hat{H}}$) is recalculated. A *P* value for each adjacency pair (entry in ${\hat{H}}$*)* is calculated as the fraction of observations that are more extreme (greater than or less than) than the ground truth value in *H*:

9 $$P = \frac{{n_{trials} - \left( {count\,of\,entries\,{\hat{H}}_{ij} > \left( < \right)H_{ij}} \right) + 1}}{{n_{trials} + 1}}$$

### Clustering for adjacency discovery

Agglomerative hierarchical clustering was performed to verify that cohorts with parameterized spatial distributions and spatial null cohorts exhibited the expected significant adjacencies and avoidances as well as to identify significant adjacencies of more than two cell types^32^. For a given in silico tissue, a permutation test was performed for each possible adjacency, and a *P* value was calculated. Adjacencies were clustered on the basis off these scores using the unweighted pair group method with arithmetic mean (UPGMA) algorithm, as implemented in Scipy v. 1.4.1.

### Adjacency enrichment statistic and *Z*-test

We defined a statistic to quantify the overall enrichment of a cell–cell adjacency relative to an expectation on the basis of the proportion of cell types and a linear algebraic method for fast computation. As a theoretical framing, consider a tissue to be an undirected graph *G*(*V*,*E*) in which cells are represented by vertices, and an edge represents a direct adjacency between two cells. The *K* types are encoded in the graph as attributes on the vertices. For an adjacency between two cells of type A and B, we define the expectation of the number of edges in the graph that connect a cell of type A and a cell of type B as:

10 $$\Sigma = 2f{_{\mathrm{A}}}\,f{_{\mathrm{B}}}\left| E \right|$$

where *f*_*k*_ is the frequency of a cell of type *k*. Then, we define the AES as:

11 $$x{_{\mathrm{AB}}} = \frac{{N{_{\mathrm{AB}}}}}{\Sigma } - 1,$$

where *N*_*AB*_ is the number of edges connecting a cell of type A with a cell of type B. An AES of 0 indicates no enrichment over expectation, negative and positive values indicate depletion and enrichment, respectively.

To conduct a test of difference between two AES distributions, we calculate:

12 $$z = \frac{{\bar X _{{AB}^{(1)}} - \bar X _{{AB}^{(2)}}}}{{\sqrt {\frac{{\sigma _{{AB}^{(1)}}}}{{\sqrt {n^{\left( 1 \right)}} }} + \frac{{\sigma _{{AB}^{\left( 2 \right)}}}}{{\sqrt {n^{\left( 2 \right)}} }}} }},$$

where $\underline X _{AB}^{\left( i \right)}$ and $\sigma _{AB}^{\left( i \right)}$are the sample mean and s.d. of AESs between cells of type A and B in sample 1. The one-sided probability of *z* (*P* value) is calculated using a standard Gaussian survival function.

The following method was devised to efficiently calculate the AES in complex graphs. Let *A* be the adjacency matrix corresponding to *G*, and *B* be a |*V*| × *K* matrix of one-hot encodings of cell type. Let *i* and *j* be the indices corresponding to the one-hot encoding of types X and Y, respectively. (A one-hot encoding in this context means that, for a row in *B*, the entry corresponding to the cell’s assigned type is 1 and all other entries in the row are 0 (all row sums are equal to 1).) The symmetric matrix $C = B^{\it{ \top }}AB$ is constructed, and the value *N*_*AB*_ is calculated in equation (11) as follows: if *i* ≠ *j*, the element *C*_*i,j*_ is equivalent to the number of edges (that is, adjacencies) between types X and Y, or if *i* = *j*, the number of edges between two cells of the same type is $\frac{{C_{i,\,j}}}{2}$.

### Retrospective power analysis

A retrospective power analysis was conducted by generating tissues with three different spatial compositions. Through a permutation test, a list of all significant adjacencies and avoidances was compiled in each generated tissue to establish a ground truth of the full diversity of spatial adjacencies in a sample. Then, contiguous spatial samples of increasing size were drawn, and a permutation test was conducted to identify significant adjacencies and avoidances within the sub-sample. The identified significant adjacencies and avoidances from the sub-sample were compared with the ground truth and the proportion of ground truth spatial adjacencies that were recovered in the sub-sample were calculated along with the proportion of falsely called significant adjacencies and avoidances in the sub-sample. For each size increment, 100 trials were conducted.

### Spatial binning to generate Visium-like data

The spatial resolution of data sets with single-cell or near-single-cell spatial resolution was reduced by creating spatial bins. Cells were grouped into ‘spatial spots’ whose centers were arrayed over the tissue in a triangular grid such that each spot center was 100 µM from all other spot centers. All cells within a 27.5-µM radius of each spot center were assigned to that spatial spot. Cells that fell outside any spatial spot radius were discarded.

### Spatial resolution analysis

To estimate sampling requirements to detected at least one cell of a specific cell type in spatially binned data, the same procedure was performed as described in the ‘Cell-type heterogeneity’ section, but to detect ‘spatial spots of interest,’ defined as any spot containing a detectable number of cells of the type of interest. We dynamically set this threshold to mimic errors in cell type deconvolution. Prior works have this error at 5–11% (refs. ^38,39^), and thus we set a per-spot threshold of 10% of cells in the simulated spot being of the type of interest. Using this threshold, we declared any spot with more than 10% of cells being of the type of interest to be a ‘spatial spot of interest.’ The model and sampling procedures remained otherwise unchanged.

### Multiple sample analysis

To compare the impact of sampling a certain number of FOVs from one or multiple tissues, FOVs of fixed size were sampled from one, two, or three tissues, owing to data availability. Sets of tissues were randomized such that all combinations of one, two, or three tissues in the data set were sampled. For each trial, the mean number of FOVs required to detect at least one cell of type of interest at 80% probability was calculated, using the sampling models for cel-type discovery previously described.

### Figure generation

All figures shown in this work were generated with Matplotlib 3.4.2.

### Reporting summary

Further information on research design is available in the Nature Portfolio Reporting Summary linked to this article.

## Online content

Any methods, additional references, Nature Portfolio reporting summaries, source data, extended data, supplementary information, acknowledgements, peer review information; details of author contributions and competing interests; and statements of data and code availability are available at 10.1038/s41592-023-01766-6.

## Supplementary information

Supplementary Information Supplementary Figures 1–9 and Supplementary Table 1

Reporting Summary

## Data Availability

All data used in this study have been previously published and are available via the respective publications. The CODEX spleen data set is available at 10.17632/zjnpwh8m5b.1 The HDST breast cancer data set is available in the Broad Institute Single Cell Portal at https://singlecell.broadinstitute.org/single_cell/study/SCP420/hdst. The osmFISH data set of mouse cortex is available at http://linnarssonlab.org/osmFISH/.

## Code availability

Code for tissue generation and power analysis is available at https://github.com/klarman-cell-observatory/PowerAnalysisForSpatialOmics and is archived in Zenodo^40^.

## References

[1] Jackson HW (2020). The single-cell pathology landscape of breast cancer. Nature.

[2] He B (2020). Integrating spatial gene expression and breast tumour morphology via deep learning. Nat. Biomed. Eng..

[3] Schürch CM (2020). Coordinated cellular neighborhoods orchestrate antitumoral immunity at the colorectal cancer invasive front. Cell.

[4] Marjanovic ND (2020). Emergence of a high-plasticity cell state during lung cancer evolution. Cancer Cell.

[5] Keren L (2018). A structured tumor-immune microenvironment in triple negative breast cancer revealed by multiplexed ion beam imaging. Cell.

[6] Ali HR (2020). Imaging mass cytometry and multiplatform genomics define the phenogenomic landscape of breast cancer. Nat. Cancer.

[7] Chen KH, Boettiger AN, Moffitt JR, Wang S, Zhuang X (2015). RNA imaging. Spatially resolved, highly multiplexed RNA profiling in single cells. Science.

[8] Lubeck E, Coskun AF, Zhiyentayev T, Ahmad M, Cai L (2014). Single-cell in situ RNA profiling by sequential hybridization. Nat. Methods.

[9] Lee JH (2014). Highly multiplexed subcellular RNA sequencing in situ. Science.

[10] Ke R (2013). In situ sequencing for RNA analysis in preserved tissue and cells. Nat. Methods.

[11] Codeluppi, S. *et al*. Spatial organization of the somatosensory cortex revealed by osmFISH. *Nat. Methods* **15**, 932–935 (2018). 10.1038/s41592-018-0175-z 30377364

[12] Ståhl PL (2016). Visualization and analysis of gene expression in tissue sections by spatial transcriptomics. Science.

[13] Rodriques SG (2019). Slide-seq: a scalable technology for measuring genome-wide expression at high spatial resolution. Science.

[14] Vickovic S (2019). High-definition spatial transcriptomics for in situ tissue profiling. Nat. Methods.

[15] Angelo M (2014). Multiplexed ion beam imaging of human breast tumors. Nat. Med..

[16] Giesen C (2014). Highly multiplexed imaging of tumor tissues with subcellular resolution by mass cytometry. Nat. Methods.

[17] Goltsev Y (2018). Deep profiling of mouse splenic architecture with CODEX multiplexed imaging. Cell.

[18] Merritt CR (2020). Multiplex digital spatial profiling of proteins and RNA in fixed tissue. Nat. Biotechnol..

[19] Chen A (2022). Spatiotemporal transcriptomic atlas of mouse organogenesis using DNA nanoball-patterned arrays. Cell.

[20] Liu Y (2020). High-spatial-resolution multi-omics sequencing via deterministic barcoding in tissue. Cell.

[21] Vickovic S (2022). SM-Omics is an automated platform for high-throughput spatial multi-omics. Nat. Commun..

[22] Butler A, Hoffman P, Smibert P, Papalexi E, Satija R (2018). Integrating single-cell transcriptomic data across different conditions, technologies, and species. Nat. Biotechnol..

[23] Schmid, K.T., Höllbacher, B., Cruceanu, C. et al. scPower accelerates and optimizes the design of multi-sample single cell transcriptomic studies. *Nat. Commun.* **12**, 6625 (2021). 10.1038/s41467-021-26779-7 10.1038/s41467-021-26779-7 PMC8595682 34785648

[24] Liang, S., Willis, J., Dou, J. et al. Sensei: how many samples to tell a change in cell type abundance? *BMC Bioinform.* **23**, 2 (2022). 10.1186/s12859-021-04526-5 10.1186/s12859-021-04526-5 PMC8728970 34983369

[25] Svensson V (2017). Power analysis of single-cell RNA-sequencing experiments. Nat. Methods.

[26] Davis A, Gao R, Navin NE (2019). SCOPIT: sample size calculations for single-cell sequencing experiments. BMC Bioinformatics.

[27] Tarmo Ä. *et al*. Splotch: Robust estimation of aligned spatial temporal gene expression data. Preprint at 10.1101/757096 (2019).

[28] Qian X (2019). Probabilistic cell typing enables fine mapping of closely related cell types in situ. Nat. Methods.

[29] Arnol D, Schapiro D, Bodenmiller B, Saez-Rodriguez J, Stegle O (2019). Modeling cell-cell interactions from spatial molecular data with spatial variance component analysis. Cell Rep..

[30] Tanevski, J., Flores, R.O.R., Gabor, A. et al. Explainable multiview framework for dissecting spatial relationships from highly multiplexed data. *Genome Biol.* **23**, 97 (2022). 10.1186/s13059-022-02663-5 10.1186/s13059-022-02663-5 PMC9011939 35422018

[31] Rajaram S (2017). Sampling strategies to capture single-cell heterogeneity. Nat. Methods.

[32] Schapiro D (2017). histoCAT: analysis of cell phenotypes and interactions in multiplex image cytometry data. Nat. Methods.

[33] Chen, Z. *et al.* Modeling Multiplexed Images with *Spatial-LDA* Reveals Novel Tissue Microenvironments. *J. Comput. Biol.* **27**, 1204–1218 (2020). 10.1089/cmb.2019.0340 PMC7415889 32243203

[34] Tanevski J, Flores ROR, Gabor A, Schapiro D, Saez-Rodriguez J (2022). Explainable multiview framework for dissecting spatial relationships from highly multiplexed data. Genome Biol..

[35] Leighton FT (1979). A graph coloring algorithm for large scheduling problems. J. Res. Natl Bur. Stand..

[36] Savani V, Zhigljavsky AA (2006). Efficient estimation of parameters of the negative binomial distribution. Commun. Stat. Theory Methods.

[37] Virtanen P (2020). SciPy 1.0: fundamental algorithms for scientific computing in Python. Nat. Methods.

[38] Miller BF, Huang F, Atta L, Sahoo A, Fan J (2022). Reference-free cell type deconvolution of multi-cellular pixel-resolution spatially resolved transcriptomics data. Nat. Commun..

[39] Biancalani T (2021). Deep learning and alignment of spatially resolved single-cell transcriptomes with Tangram. Nat. Methods.

[40] Baker, E. & Schapiro, D. *klarman-cell-observatory/PowerAnalysisForSpatialOmics: publication_archive_20221128*. (2022); 10.5281/zenodo.7372872

