# Supplementary material for: In silico tissue generation and power analysis for spatial omics
Source: Nat Methods. 2023 Mar 2;20(3):424–31. doi: 10.1038/s41592-023-01766-6 (PMC9998272; doi:10.1038/s41592-023-01766-6)
Supplement: Supplementary file 1 — Supplementary Figures 1–9 and Supplementary Table 1 [file 41592_2023_1766_MOESM1_ESM.pdf]

nature methods

Article

https://doi.org/10.1038/s41592-023-01766-6

In silico tissue generation and power analysis for spatial omics

In the format provided by the authors and unedited

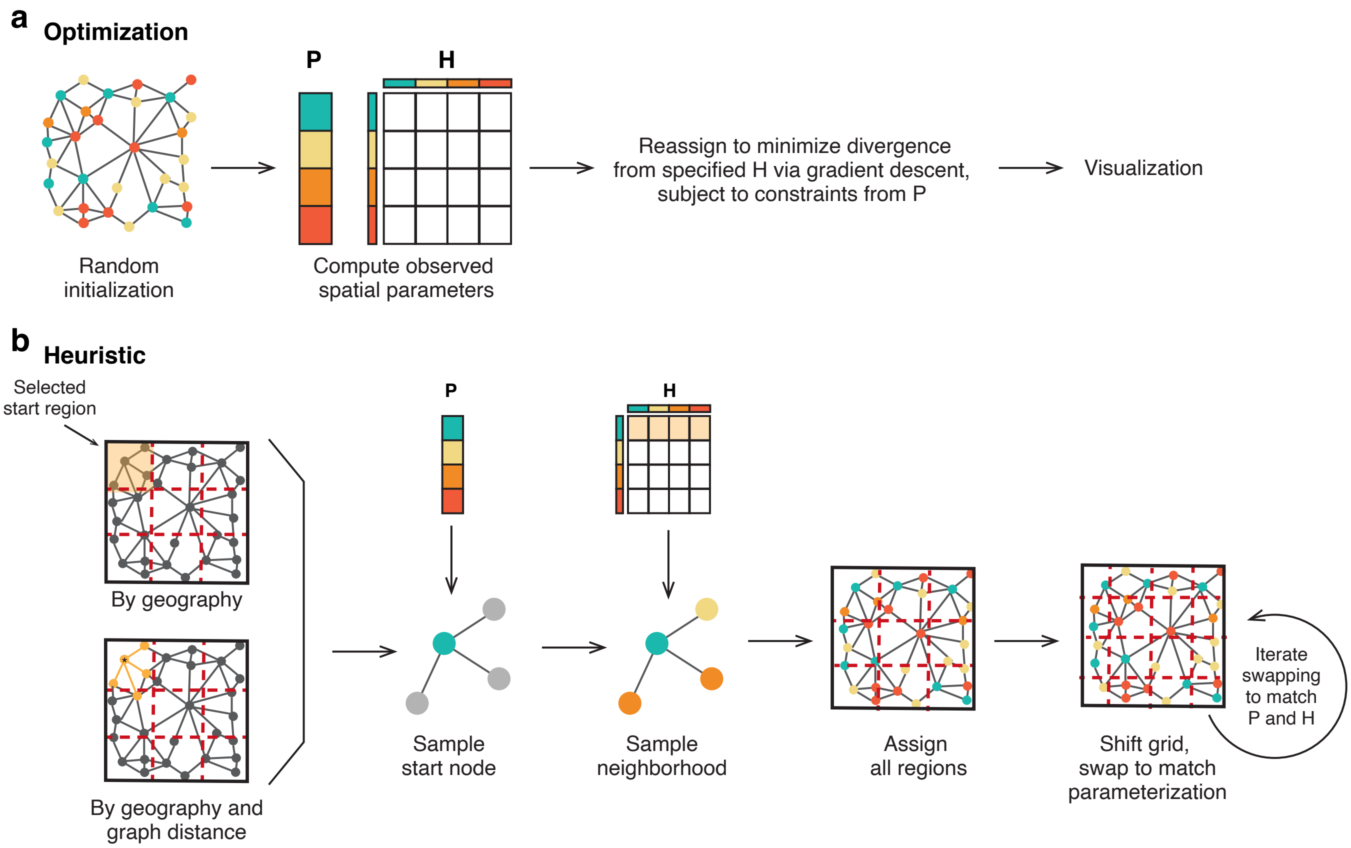

Supplementary Fig. 1: Labeling tissue scaffolds
(a) Overview of methods used to optimize tissue scaffold labeling. Spatial parameters (biological priors) and the tissue scaffold are provided to the assignment algorithm, which optimizes cell type labeling on the scaffold via gradient descent. **(b)** Overview of the heuristic solution used to optimize tissue scaffold labeling. Spatial parameters (biological priors) and the tissue scaffold are provided to the assignment algorithm, which attempts to generate a labeling that matches specified spatial parameters.

Supp. Fig. 2

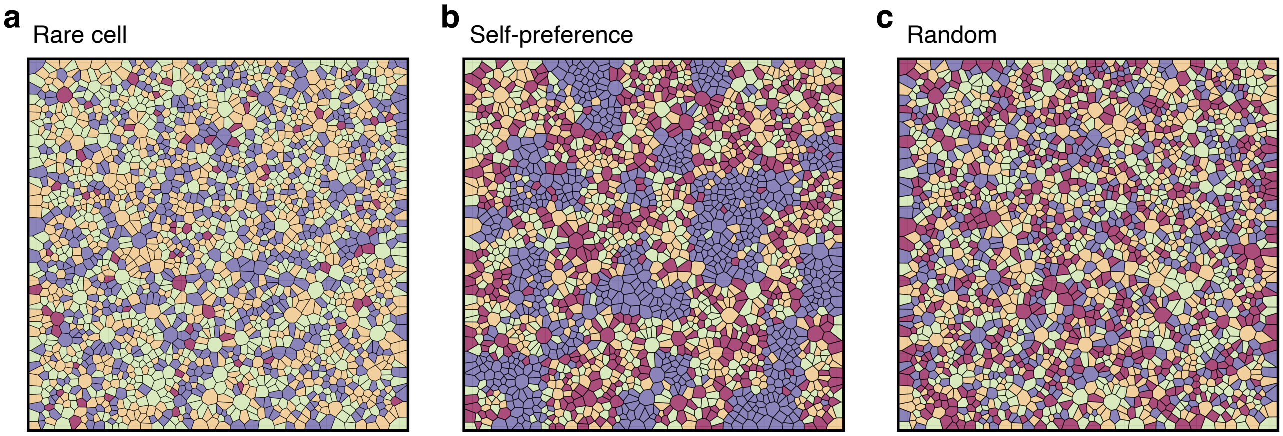

Supplementary Fig. 2: Example in silico tissues mimicking three biological scenarios. ISTs illustrating tissues with a rare cell type (**a**, maroon), one cell type with a strong preference to locate next to cells of the same type (**b**, purple), and no spatial constraints (**c**). Other cell types (colors) are of roughly equal abundance and have random distribution in space.

Supp. Fig. 3

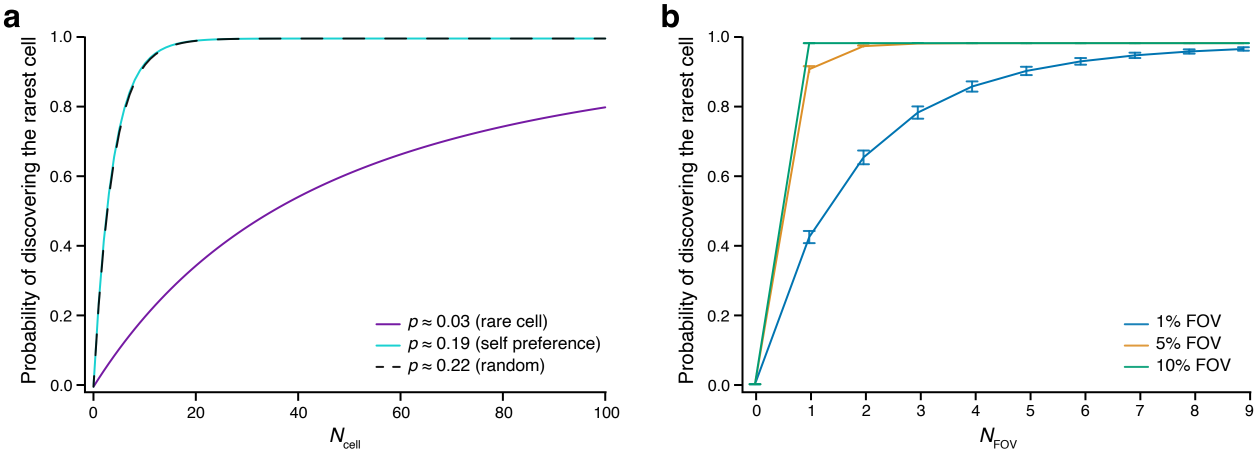

Supplementary Fig. 3: Effect of cell type abundance and FOV size on cell type detection in small in silico tissues.

(**a**) Impact of number of sampled cells on rare cell type detection. Beta-binomial probability (y axis) that the rarest cell type is observed at least once when sampling different numbers of cells (x axis, N_cells), for each of the three in silico tissue types in **Supplementary Fig. 2**. (**b**) Impact of size of FOV on rare cell type detection. Beta-binomial probability (y axis) that the rarest cell type is observed in at least one FOV when sampling different numbers of FOVs (x axis), for FOVs of sizes equivalent to 1% (blue), 5% (orange), or 10% (green) of total tissue size. Error bars indicate a 95% confidence interval calculated over 100 independent experiments (20 independent FOVs drawn at each size per experiment).

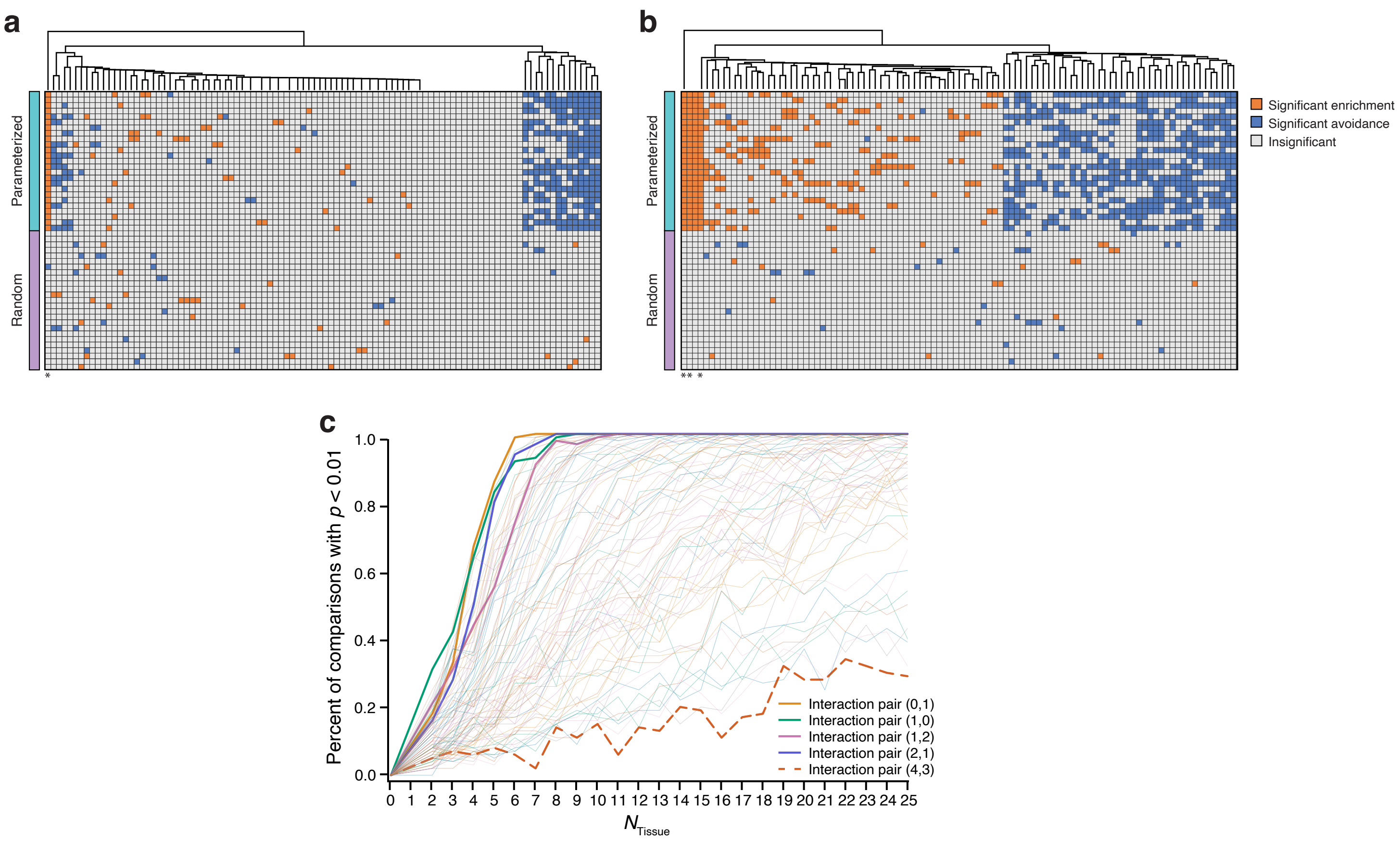

Supplementary Fig 4: Significant pairwise adjacencies between cell types can be generated and detected in in silico data.
(**a,b**) Significant pairwise adjacencies recovered in parametrized ISTs. Statistically significant ($p<0.01$, permutation test, one-sided) avoidances (blue) and adjacencies (orange) between each pair of cell types (columns) (10 cell types, 100 pairs) in each of 50 ISTs (rows), 25 with random spatial pattern (spatial null, purple), and another 25 IST parametrized (cyan) with either a self-preference for one cell type (**a**) or with a significant motif of 3 adjacent cell types. "*" denotes the specified significant adjacency pair. (**b**). The self-preference adjacency in (**a**) is in the leftmost row. "*" denotes the specified significant adjacency pairs. (**c**) Impact of number of tissues on detection of significant adjacencies. Fraction of comparisons returning a statistically significant ($p < 0.01$, two-sided T test) difference (y axis) between parameterized IST (with a significant 3 cell types adjacency) and null ISTs (10 cell types per IST), for different numbers of ISTs per category (x axis). Adjacency pairs (0,1), (1,0), (1,2), and (2,1) (highlighted traces) are the parameterized cell types representing the adjacency of interest. Adjacency pair (4,3) (dashed line) was not a specified relationship in the tissue generation. Transparent traces show results for adjacency pairs that were not specified to be enriched in the tissue generation.

Supp. Fig. 5

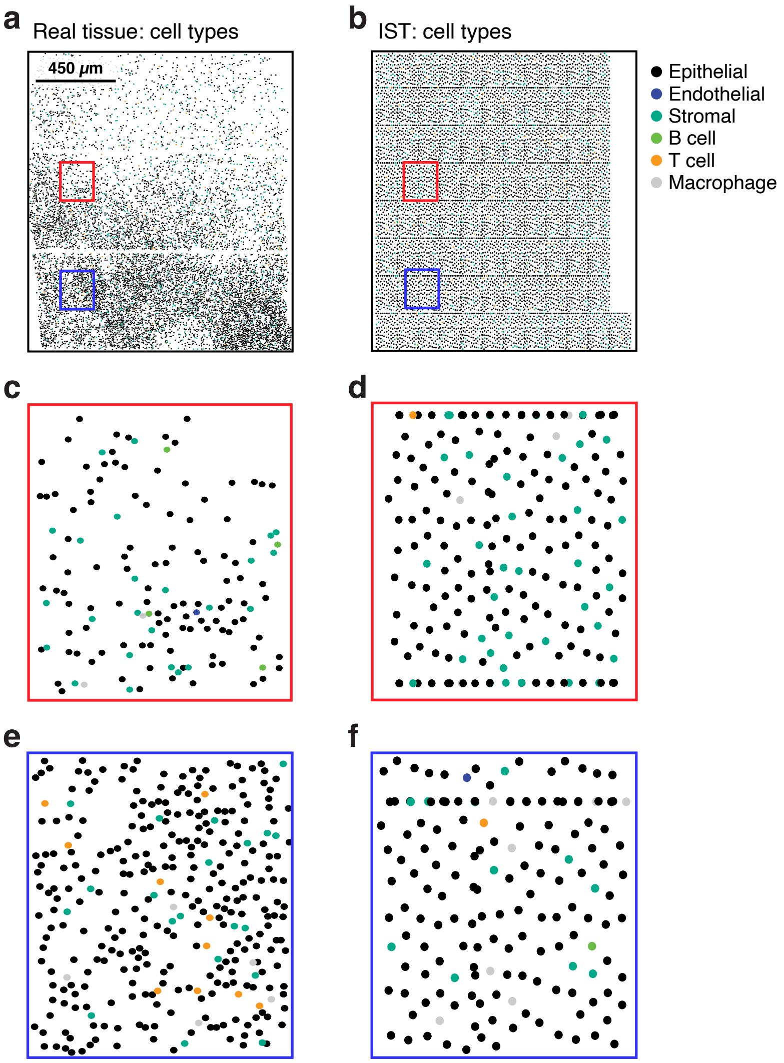

Supplementary Fig. 5: Breast cancer ISTs mimic real spatial data.
(**a,b**) Parameters from real HDST data in breast cancer are used to generate an IST. Cells (dots) colored by cell type label in real HDST (**a**) and the ISR generated based on its spatial parameters (**b**). Red and blue boxes are matching regions in HDST and the IST expanded in (**c-f**). (c-f) Agreement in region characteristics between HDST and the generated IST. An expanded view of the red (**c,d**) or blue (**e,f**) regions in HDST (**a**) or the IST (**b**). The red region in both HDST (**c**) and IST (**d**) is dominated by epithelial cells (black) with relatively high occurrence of stromal cells (teal). The blue regions in both HDST (**e**) and IST (**f**) is also dominated by epithelial cells (black) but with a lower proportion of stromal cells (teal), and a higher abundance of T cells (orange) and macrophages (grey), as well as B cells (green) in the IST (**e**) but not HDST (**f**).

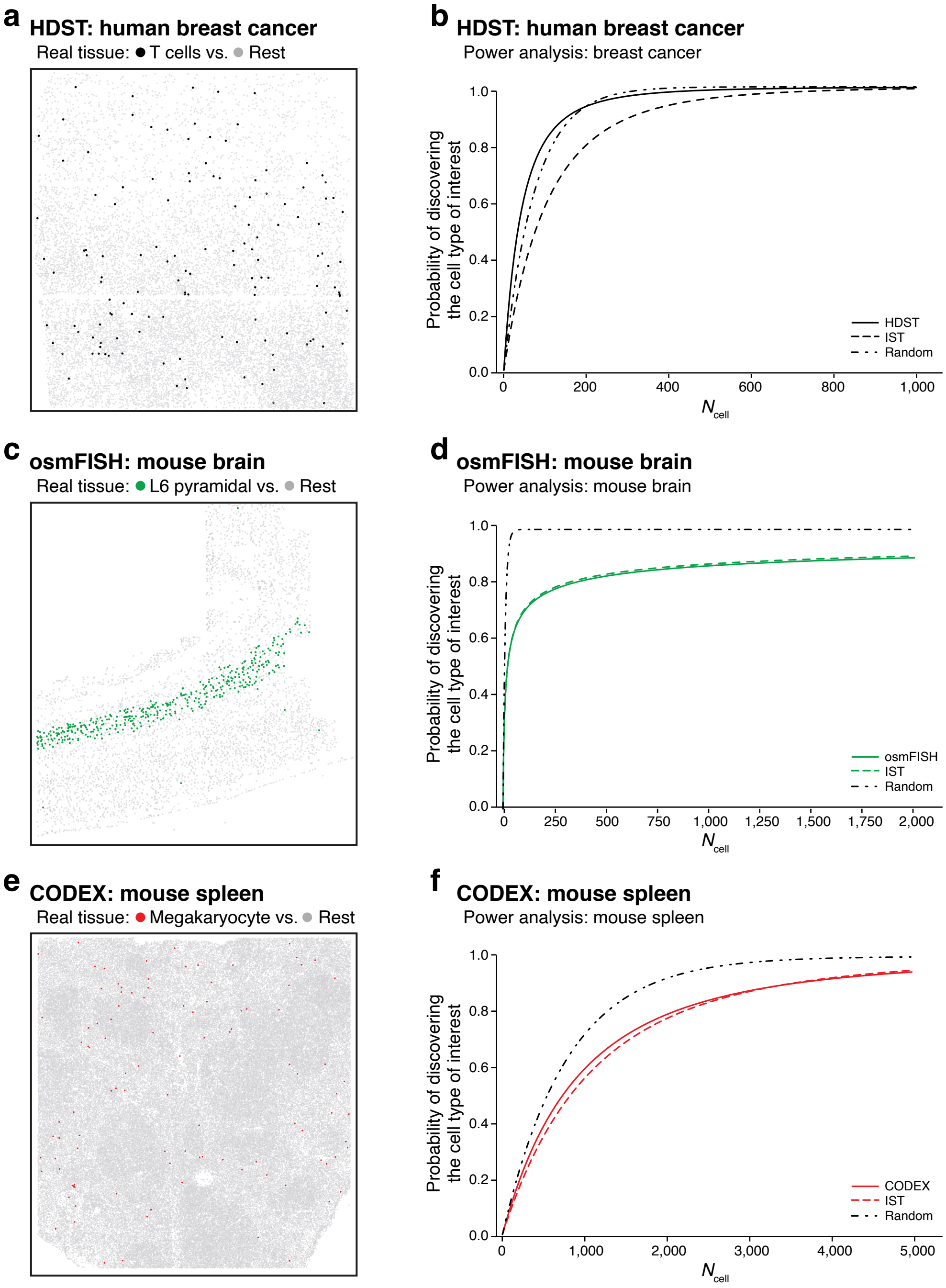

Supplementary Fig 6.: Power analysis for single cell sampling for cell type detection. (**a, c, e**) Cell type (color) for detection in real spatial data from breast cancer (**a**, T cell), mouse cortex (**c**, L6 pyramidal neurons) and spleen (**e**, megakaryocytes) spatial data. (**b,d,f**) Impact of number of sampled cells to detect selected cell types from tissues with different structures. Probability of detecting the highlighted cell type (y axis) when sampling different numbers of cells via a spatial sampling strategy ("regional sampling") real datasets of breast cancer (**b**), mouse cortex (**d**) and spleen (**f**) (solid lines) and from ISTs parameterized based on breast cancer (**b**), mouse cortex (**d**) and spleen (**f**) data (dashed lines), or if the selected cell line was randomly distributed in the tissue at its observed frequency in the dataset (dashed-dotted line).

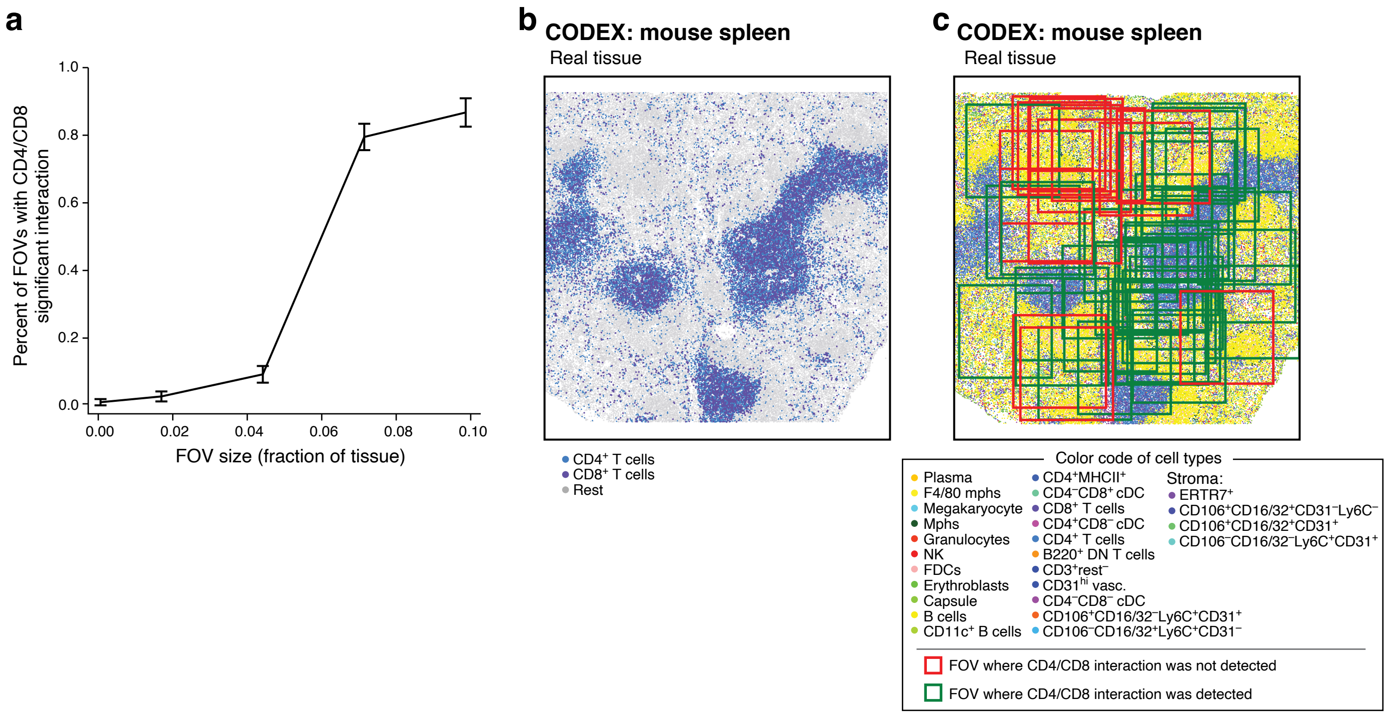

Supplementary Fig. 7: Effect of FOV size on the detection of a significant CD4+/CD8+ T cell adjacency in mouse spleen

(**a**) Impact of FOV size on probability to detect cell-cell adjacency. The fraction of FOVs with a significantly enriched CD4+/CD8+ T cell adjacency (y axis) for different FOVs sizes (x axis, % of total tissue size). Error bars indicate a 95% confidence interval, calculated over 100 independent FOVs at each size. (**b,c**) CD4+ CD8+ T cell adjacencies. (**b**) CODEX data with cells (dots) colored by CD4+ (light blue) and CD8+ (dark blue) labels. (**c**) FOV selections (squares), each sized at 7.5% of total tissue area, where CD4+ CD8+ T cell adjacencies were significantly enriched (green) or not (red).

Supp. Fig. 8

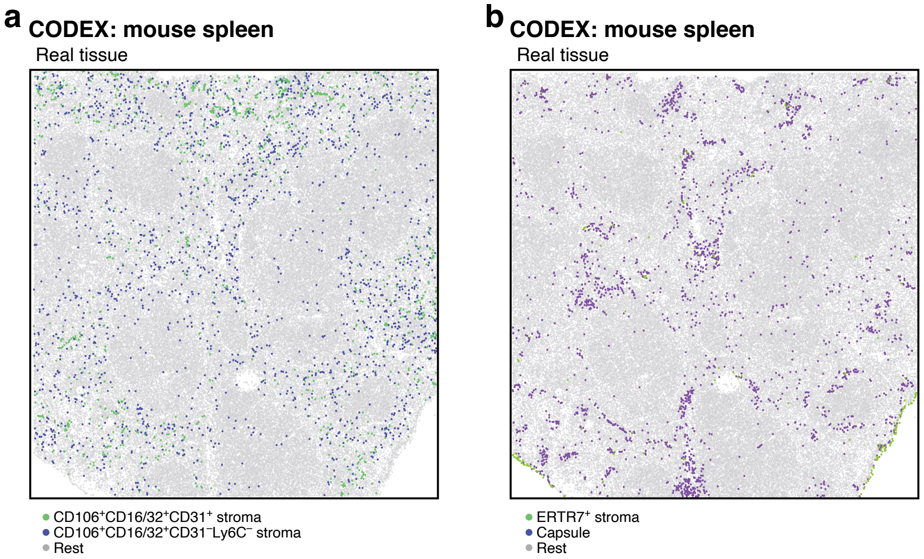

Supplementary Fig. 8: Cells participating in significant adjacencies that were not detected in the IST cohort are at the margins of the real tissue.

Cells (dots) labeled by cell type (color) in real spleen CODEX data for a significant adjacency detected by IST analysis (**a**, between CD106+CD16/32+CD31+ and CD106+CD16/32+CD31-Ly6C- cells), and for a significant adjacency not found in ISTs (**b**, between ERTR7+ cells and the capsule at the tissue edges).

Supp. Figure 9

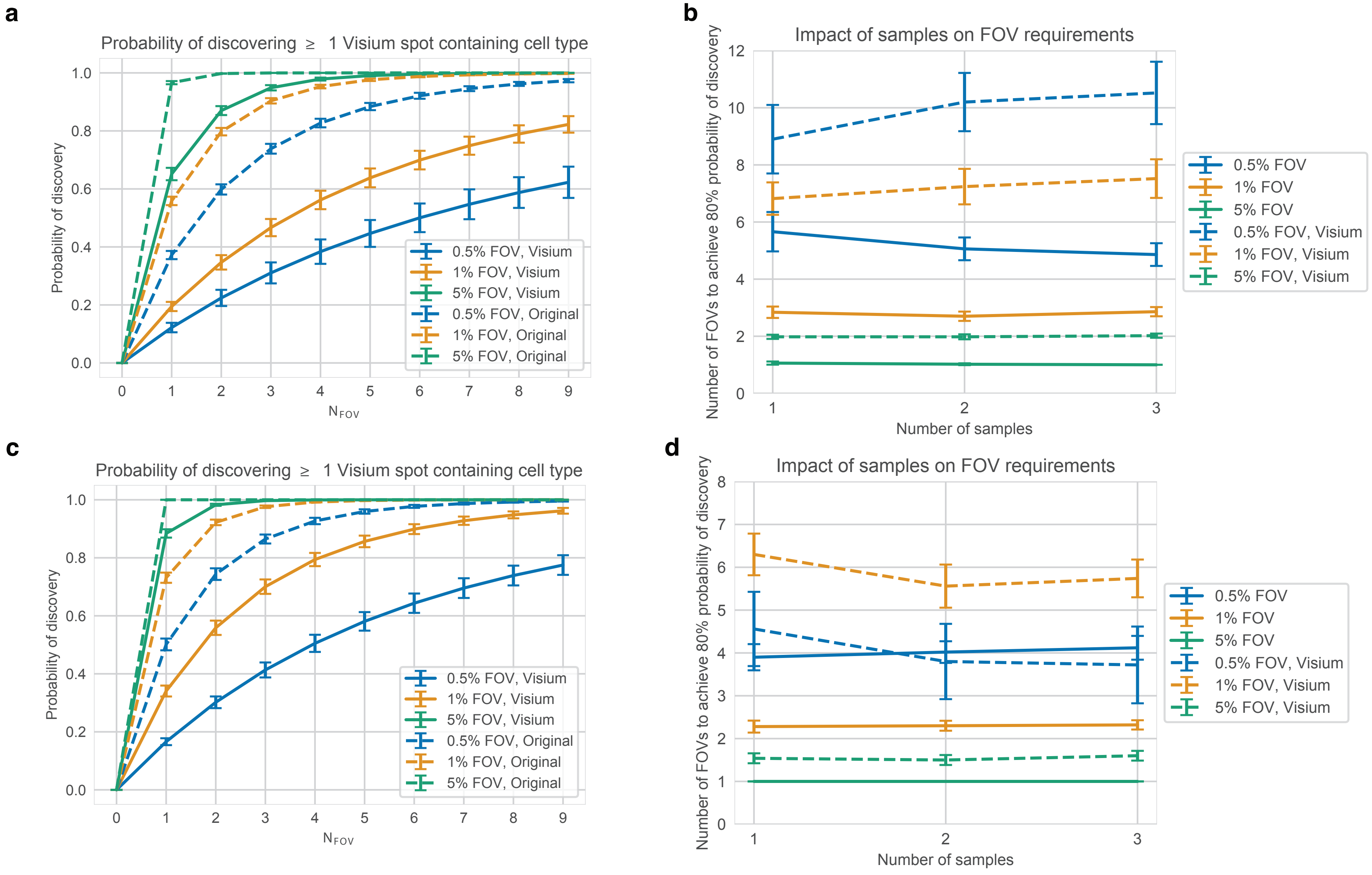

Supplementary Figure 9: Impact of spatial resolution and multiple tissues on spatial power
(**a,c**) Lower spatial resolution reduces power. Probability (y-axis) to detect at least one Visium spot containing at least one cell with a cell type of interest (**a**, megakaryocyte; **c**, T cell), or at least one cell (from native resolution, as in **Fig. 2**) when sampling different numbers of FOVs (x-axis) of different sizes (solid lines) in CODEX spleen (**a**) or HDST breast (**c**) data. (**b,d**) Sampling FOVs from different tissues does not impact detection power. The expected number of FOVs (y-axis) required to detected at least one cell (**b**, megakaryocyte in original CODEX data; **d**, T cell in original HDST data, solid lines) or at least one containing spot (in Visium-like data, dashed lines) at 80% probability when FOVs are drawn from one, two, or three distinct tissue samples (x-axis) for FOVs of different sizes (colored lines). (**a-d**) Error bars indicate a 95% confidence interval calculated over 50 independent simulations (30 independent FOVs drawn at each size per experiment).

Supplemental Table 1: Summary of experimental methods for spatial data generation

**Transcriptomic**	*Sequencing*	Slide-seq[1]
		Spatial Transcriptomics (commercially available as 10X Visium)[2]
		High-definition spatial transcriptomics (HDST)[3]
		In situ RNA sequencing[4]
	Hybridization	cyclic-ouroboros smFISH (osmFISH)[5]
		sequential Fluorescence In Situ Hybridization (seqFISH)[6]
		multiplexed error-robust FISH (merFISH)[7]
**Proteomic**	*Imaging*	CO-Detection by indEXing (CODEX)[8]
		Cyclic Immunofluorescence (CyCIF) [9,10]
		Multiplexed immunohistochemical consecutive staining on single slide (MICSSS)[11]
	Mass spectrometry	Imaging Mass Cytometry (IMC)[12]
		Multiplexed Ion Beam Imaging (MIBI)[13]

References

1. Rodriques, S. G. *et al.* Slide-seq: A scalable technology for measuring genome-wide expression at high spatial resolution. *Science* **363**, 1463–1467 (2019).
2. Ståhl, P. L. *et al.* Visualization and analysis of gene expression in tissue sections by spatial transcriptomics. *Science* **353**, 78–82 (2016).
3. Vickovic, S. *et al.* High-definition spatial transcriptomics for in situ tissue profiling. *Nat. Methods* **16**, 987–990 (2019).
4. Ke, R. *et al.* In situ sequencing for RNA analysis in preserved tissue and cells. *Nat. Methods* **10**, 857–860 (2013).
5. Codeluppi, S. *et al.* Spatial organization of the somatosensory cortex revealed by osmFISH. *Nature Methods* vol. 15 932–935 (2018).
6. Eng, C.-H. L. *et al.* Transcriptome-scale super-resolved imaging in tissues by RNA seqFISH. *Nature* vol. 568 235–239 (2019).
7. Chen, K. H., Boettiger, A. N., Moffitt, J. R., Wang, S. & Zhuang, X. RNA imaging. Spatially resolved, highly multiplexed RNA profiling in single cells. *Science* **348**, aaa6090 (2015).
8. Goltsev, Y. *et al.* Deep Profiling of Mouse Splenic Architecture with CODEX Multiplexed Imaging. *Cell* **174**, 968–981.e15 (2018).
9. Lin, J.-R., Fallahi-Sichani, M. & Sorger, P. K. Highly multiplexed imaging of single cells using a high-throughput cyclic immunofluorescence method. *Nat. Commun.* **6**, 8390 (2015).
10. Lin, J.-R. *et al.* Highly multiplexed immunofluorescence imaging of human tissues and tumors using t-CyCIF and conventional optical microscopes. *Elife* **7**, (2018).
11. Remark, R. *et al.* In-depth tissue profiling using multiplexed immunohistochemical consecutive staining on single slide. *Sci Immunol* **1**, aaf6925 (2016).
12. Giesen, C. *et al.* Highly multiplexed imaging of tumor tissues with subcellular resolution by mass cytometry. *Nat. Methods* **11**, 417–422 (2014).

13. Angelo, M. *et al.* Multiplexed ion beam imaging of human breast tumors. *Nat. Med.* **20**, 436–442 (2014).
